# Hemocompatibility of Mechanical Circulatory Support Devices: An Integrated Review of Platelet Function Assessment in In-Vitro Blood Experiments

**DOI:** 10.1007/s10439-026-04173-y

**Published:** 2026-06-04

**Authors:** Xiangyu He, Michael B. Fischer, Daniel Zimpfer, Alice Assinger, Marcus Granegger

**Affiliations:** 1 Christian Doppler Laboratory for Mechanical Circulatory Support, Department of Cardiac and Thoracic Aortic Surgery, Medical University of Vienna, AKH Wien, Währinger Gürtel 18-20, A-1090 Wien, Vienna, Austria; 2 Clinic for Transfusion Medicine and Cellular Therapy, Medical University of Vienna, Vienna, Austria; 3 Department for Biomedical Research, Danube University Krems, Krems, Austria; 4 Ludwig Boltzmann Institute of Cardiovascular Research, Vienna, Austria; 5 Institute of Vascular Biology and Thrombosis Research, Center for Physiology and Pharmacology, Medical University of Vienna, Vienna, Austria

**Keywords:** Mechanical circulatory support device, Platelet function, in vitro experiments, Shear stress, Blood experiment

## Abstract

**Purpose:**

Despite persistent bleeding and thrombotic adverse events in patients supported with blood pumps, standardized preclinical assessment of hemocompatibility remains largely restricted to hemolysis testing. To advance the field, robust and comparable in vitro platelet function testing within mock-loop mechanical circulatory support (MCS) studies is needed. This review systematically summarizes current methodologies for platelet function assessment in MCS devices and critically examines their advantages and limitations to inform the development of a unified, evidence-based testing framework.

**Methods:**

A systematic literature search was conducted in PubMed, Web of Science, Embase, and Scopus using Medical Subject Headings (MeSH) and free-text terms in accordance with PRISMA guidelines. Articles were screened according to predefined inclusion and exclusion criteria.

**Results:**

Thirty-nine studies met the eligibility criteria. Study designs varied substantially with respect to experiment duration, sampling strategies, mock-circulation loop configurations, and pump types. Human whole blood was the predominant test medium (*n* = 27). Anticoagulation strategies differed widely, with citrate-based formulations (*n* = 22) and heparin (*n* = 15) most frequently employed. Flow cytometry was the predominant analytical method (*n* = 25), primarily assessing platelet function through changes in surface receptor expression or shedding.

**Conclusion:**

Flow cytometry offers a highly informative and direct approach for assessing platelet state in MCS research compared to other methods. However, substantial methodological heterogeneity continues to hinder data interpretation and cross-study comparability. Beyond standardizing experimental setups and protocols, future investigations should incorporate multiple complementary biomarkers and clarify their relationships to clinically relevant outcomes to more comprehensively characterize shear-induced platelet responses.

## Introduction

Mechanical circulatory support (MCS), particularly left ventricular assist devices (LVADs), has become an established therapeutic option for patients with advanced heart failure [1], enabling restoration of cardiac output and end-organ perfusion in cases where conventional therapies are insufficient. Contemporary MCS devices predominantly rely on continuous-flow rotary pumps, which operate using rapidly spinning impellers to propel blood and narrow flow pathways. While these designs have improved durability and clinical outcomes, they inherently expose blood components to non-physiological flow conditions, characterized by supraphysiological shear stresses and complex flow patterns such as recirculation zones and regions of flow stagnation [2, 3].

These mechanical forces, in combination with the time blood particles are exposed to them (residence time), play a central role in blood damage [3]. Shear-mediated alterations affect multiple blood components, including hemolysis of erythrocytes, degradation of von Willebrand factor (vWF), alteration of platelets function, and leukocyte activation [1]. For example, supraphysiological shear can unfold vWF multimers, rendering them susceptible to cleavage by ADAMTS13 and contributing to acquired von Willebrand syndrome, a condition strongly associated with bleeding complications in LVAD-supported patients [4]. At the same time, shear-induced platelet alterations may contribute to both thromboembolic and bleeding manifestations, reflecting a complex and not yet fully understood bidirectional dysfunction of platelet activity.

Clinically, these blood-device interactions are closely linked to hemocompatibility-related adverse events (HRAEs), including bleeding and thromboembolism, which remain a major limitation of current MCS therapy despite significant technological advances [5, 6]. For example, the MOMENTUM 3 trial demonstrated improved outcomes with centrifugal-flow pumps compared to axial-flow devices, including reduced bleeding rates (45.8% vs. 56.8%) and a markedly lower incidence of suspected pump thrombosis (2.1% vs. 17.6%) [6], yet these complications were not eliminated. These observations highlight further improvements in device hemocompatibility require a deeper mechanistic understanding of shear-induced blood damage.

Comprehensive preclinical hemocompatibility assessment is therefore essential for both device development and regulatory approval. Currently, standardized evaluation is largely limited to hemolysis testing, for which established protocols exist (e.g., ASTM guidelines) [7]. In contrast, no standardized framework is available for assessing platelet function in vitro, despite its central role in both thromboembolic and bleeding complications. Existing studies employ a wide range of experimental approaches, with differences in anticoagulant choice, mock circulations, sampling procedures, hemostatic and thrombotic parameters employed and evaluation methods. These variations may significantly influence observed platelet responses [8], underscoring the need for more consistent experimental design and reporting.

As a first step towards standardization, the aim of this review is to compare existing protocols for assessing platelet function in vitro under MCS device-induced shear conditions and to identify approaches most suitable for its assessment. Ultimately, establishing an evidence-based and standardized testing framework will be essential for improving the predictive value of preclinical studies and advancing the hemocompatibility of next-generation MCS devices.

## Method

### Data Collection

This integrated review is intended to give an overview of in vitro platelet function assessment with MCS devices. This analysis was conducted in accordance with systematic review and meta-analysis (PRISMA) guidelines [9]. A systematic review of data were performed from inception to September of 2025 through the PubMed database, Embase, Web of Science and Scopus.

## Search Strategy

The following search keywords were used: ("Platelet" OR "Platelet activation" OR "Platelet aggregation" OR "Platelet function" OR "Hemocompatibility") AND ("Mechanical circulatory support" OR "Mechanical circulatory assist devices" OR "MCS" OR "Ventricular assist devices" OR "VAD" OR "Total artificial heart" OR "Blood pump" OR "Centrifugal pump" OR "Axial flow pump" OR "LVAD" OR "Left ventricular assist device" OR "Pulsatile flow device" OR "ECMO" OR "Extracorporeal circulation") AND ("Shear" OR "Shear stress" OR "Shear rate" OR "Hemodynamic stress" OR "Flow-induced"). The search was limited to original research papers. After the initial search, a screening and selection of titles and abstracts was conducted after the detection and removal of duplicates. Full articles were obtained for eligibility assessment when a study was deemed relevant. Subsequently, citation and secondary source were also checked for eligibility. Studies were selected by two independent reviewers.

## Inclusion/Exclusion Criteria

**Fig. 1:**
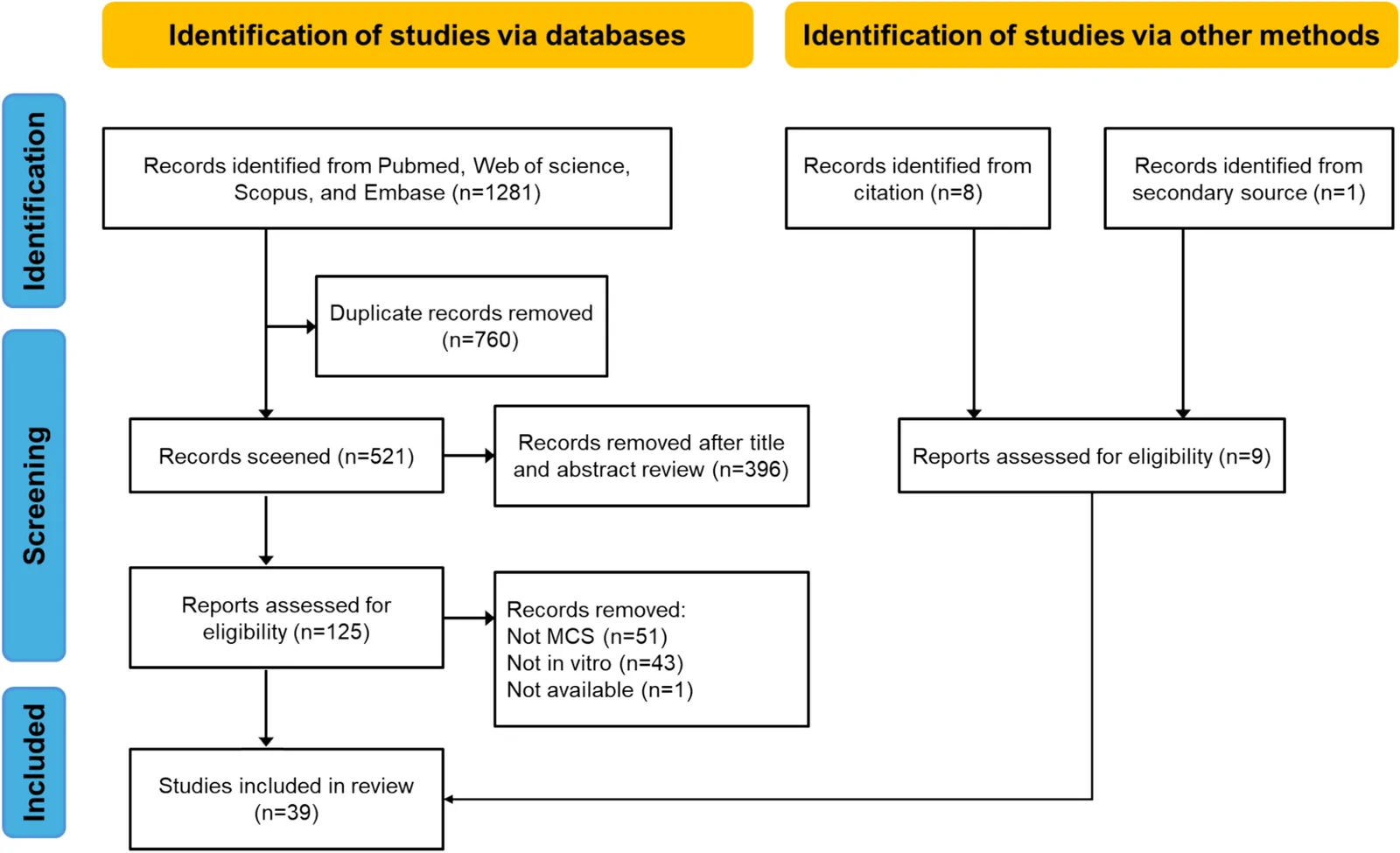
Flow diagram of the literature search according to PRISMA guidelines

Research articles were evaluated to meet the following inclusion criteria: (1) study focus is platelet function under MCS devices in mock circulating loops, (2) samples are collected from in vitro blood experiments, and (3) methods to evaluate the platelet function are described and results are presented. Articles were excluded if an English translation of the research was unavailable. A flow diagram summarizing the stepwise process of the literature search is presented in Fig. 1.

## Results

Our search strategy generated a total of 1281 results from all the different databases listed above. 760 duplicates were found and excluded. We exported these articles to Endnote to screen the studies by title and abstract. 125 articles were selected for full-text eligibility, of which 30 articles were found to meet inclusion criteria, with further 9 articles being included based on citation and secondary source searches. A total of 39 articles met the requirements to be included in this review (Fig. 1). All of these studies are summarized in Table 1.

**Table 1:** Summarization of the articles included in this review

References	Number of donors, blood type	Anticoagulants	MCS devices	Experiment duration (Sample collection)	Fixation (Lysis buffer)	Methods (Biomarkers)	Results
Zhang et al. [10]	N = 17, whole blood (human)	3.8% sodium citrate	CentriMag, CH-VAD, HVAD, HMII	4 h (every 1 h)	- (-)	Flow cytometry (CD41/61, CD42b, GPVI)	CD41/61 activation levels increased after 4-hours circulation. The MFI of antibodies bound to CD42b and GPVI receptors on platelet surface was decreased after circulation.
Wang et al. [11]	N = 7, whole blood (human)	3.2% sodium citrate	CentriMag	4 h (every 1 h)	1% PFA (-)	Flow cytometry (CD41/61, CD41)	The percentage of platelets expressing CD41/61 decreased with increasing pump pressure head and circulation time. A fairly good linear correlation between platelet CD41/61 receptor shedding and shear stress (R^2^ = 0.840)
Tarafder et al. [12]	N = 1, whole blood (human)	3.8% sodium citrate	HMII, HM3, BrioVAD	4 h (every 1 h)	- (-)	Flow cytometry (CD62P, CD42b, GPVI)	The level of CD62P increased in all pumps but highest in HMII after circulation. The levels of CD42b, GPVI consistently decreased in HMII, approximately twice as much as with HM3 and BrioVAD.
Sun et al. [13]	N = 6, whole blood (human)	3.2% sodium citrate	CentriMag Pediatric oxygenator	4 h (every 1 h)	1% PFA (-)	Flow cytometry (CD41/61, CD41, CD42b, GPVI), Fluorescence microscopy	The levels of CD41/61 activation exhibited an increasing trend with circulation time while the surface CD42b and GPVI decreased. The platelets exhibited increased adhesion capacity to fibrinogen and decreased adhesion capacities on VWF and collagen with circulation time.
Roka-Moiia et al. [14]	N = 6, GFPs (human)	ACD-A	HeartAssist-V	1 h (0, 2, 5, 10, 30, 60 min)	3.5% PFA (-)	Flow cytometry (CD41, CD62P, CD41/61, PS), PAS assay	Platelet exposure to continuous shear stress resulted in a significant increase of PS surface expression and mild CD62P exposure, while no CD41/61 activation occurred. Shear-mediated PS surface expression was significantly decreased by chelation of extracellular calcium, while intracellular calcium depletion had no significant effect. Platelet shear exposure induced thrombin generation on the platelet surface.
Roka-Moiia et al. [15]	N = 3, whole blood (porcine)	CPDA-1 (heparin 200 U/kg for porcine)	Impella 5.5, Centrimag	4 h (every 30 min)	1% PFA (-)	Flow cytometry (CD62P, PS), ELISA (soluble CD62P, β-TG)	A minor downregulation of platelet surface CD62P was detected in both devices after 4 h. After 4 h, concentration of β-TG remained essentially unchanged. The level of soluble CD62P in porcine plasma was always below the detection limit of the commercial kit (0.2 ng/ml).
Radley et al. [16]	N = 17, whole blood (bovine)	CPDA-1	CentriMag, HMII, HVAD	6 h (0, 5, 120, 240, 360 min)	Streck cell preservative (-)	Flow cytometry (CD41/61)	Activated platelets (noted as CD41/61-negative events) was elevated significantly in the HVAD at 240 and 360 minutes (P < 0 .017), and the HMII at 240 and 360 minutes (P < 0.0001) compared with the static control.
Chen et al. [17]	N = 8, whole blood (human)	Sodium citrate	CentriMag	4 h (every 1 h)	1% PFA (-)	Flow cytometry (CD41/61, GPVI, CD42b, CD41)	Levels of platelet CD41/61 and platelet receptor shedding (GPVI and CD42b) increased with circulation time, consistently higher with higher pressure heads.
Chen et al. [18]	N = 7, whole blood (human)	3.2% sodium citrate	CentriMag	4 h (every 1 h)	1% PFA (-)	Flow cytometry (CD41/61, GPVI, CD42b, CD41), ELISA (soluble CD62P)	CD41/61 activation increased over time. The pump caused progressive platelet receptor loss of GPVI and CD42b with blood circulating time. The increase in the concentration of soluble CD62P became statistically significant after two hours compared to the baseline (P < 0.05).
Chan et al. [19]	N = 6, whole blood (human)	CPDA-1 (Samples with EDTA)	HVAD	5 h (0, 60, 300 min)	BD stabilizing fixative (BD FACS lysing solution)	Flow cytometry (CD62P, CD42b)	CD62P showed significant increase in platelet activation at T = 60 min and T = 300 min, when compared with T = 0 min (P < 0.001). CD42b showed a significant increase in platelet activation only at T = 300 min compared with baseline.
Chan et al. [20]	N = 4, whole blood (bovine)	CPDA-1	EXCOR, HMII	6 h (every 2 h)	BD stabilizing fixative (BD FACS lysing solution)	Flow cytometry (CD41/61)	There was a steady increase in platelet activation (noted as CD41/61-negative events), with a statistically significant difference between 5 and 360 min for both EXCOR and HMII (P < 0.05).
Berk et al. [21]	N = 7, whole blood (human)	3.8% sodium citrate	CH-VAD, HVAD, HMII	4 h (every 1 h)	1% PFA (-)	Flow cytometry (CD41/61, CD41)	CD41‐positive events were considered as the population for analysis of platelet activation. The percentage of CD41/61 positive platelets increased with circulation time in all loops of the CH‐VAD, the HVAD, and the HMII.
Tanaka et al. [22]	N = 8 whole blood (human)	3.2% sodium citrate	Capiox centrifugal pump	3 h (every 1 h)	- (-)	Laser-light scattering assay, optical density method	The laser-light scattering method showed that generation of small and medium aggregates was not suppressed during pumping, but that generation of large aggregates was significantly reduced at 2 and 3 h of pumping. The optical density measurement showed significant reduction in platelet aggregation.
Roberts et al. [23]	N = 5 whole blood (porcine)	0.75 U/mL heparin + boluses (samples with citrate or EDTA)	Novalung Console centrifugal pump	6 h (every 3 h)	- (BD lyse wash assistant instrument)	Flow cytometry (CD61, CD62P, PS), Impedance aggregometry, SEM	Percentage of CD62P-positive and PS-positive platelets significantly increased over time. Platelet aggregation was visually reduced over time. Thrombus deposition and platelet adhesion visualized by SEM were drastically reduced.
Rauch et al. [24]	N = 16 whole blood (human)	Heparin, Tirofiban, Apyrase or EDTA	Rotaflow ECMO	3 h (0, 30, 60, 180 min)	- (-)	Flow cytometry (CD42b, GPVI)	A time-dependent decrease of platelet CD42b surface expression was observed at both low- and high-flow rates. GPVI shedding also occurred in the ECMO loop model, was more pronounced at high-flow rate.
Oota-Ishigaki et al. [25]	N = 3 whole blood (porcine)	3.2% sodium citrate (4 mL heparin + 20 mL CaCl_2_ before the start)	Centrifugal pump prototypes	1 h (every 2 min within first 10 mins, later every 10 min)	- (-)	SPF aggregometer, Photographed image, SEM	The platelet clogging on the micromesh filter occurred when the average PATI > 0.28 and the average NIH (g/20 min) > 0.01 but suppressed when PATI < 0.17 and the NIH (g/20 min) < 0.003. The average adhesion rate in all tests reached above 55%. SEM observations showed that the clogging was caused by platelets, and the degree correlates with the flow rate change.
Girdhar et al. [26]	N = 10 GFPs or GFPs + RBCs (human)	Citrate	DeBakey, HeartAssist 5	30 min (every 10 min)	- (-)	PAS assay	In both cases, platelet activation increased over time, respectively. The slope of the platelet activation linear fit in DeBakey was an order of magnitude higher than HeartAssist 5.
Consolo et al. [27]	N = 8 GFPs (ovine)	CPDA-1	Kids D100 roller pump	2 h (0, 10, 20, 30, 60, 90, 120 min)	- (-)	PAS assay	A linear evolution of platelet activation with increasing circulation time (i.e., number of passes) was observed for the GFPs recirculated.
Arao et al. [28]	N = 7 whole blood (human)	4 U/mL heparin	Stokert roller pump	3 h (0, 30, 60, 120, 180 min)	1% PFA (-)	Flow cytometry (CD41, CD42b, PS, CD62P), TEM, Fluorescence microscopy, Light transmission aggregometry	The expressions of CD42b and CD41 significantly decreased after recirculation. After recirculation the expression of CD62P was significantly lower in CD42b − platelets than in CD42b + platelets, but PS exposure in CD42b − platelets was higher than in CD42b + platelets. Platelet size after circulation was significantly smaller than before circulation. Dense bodies and α-granules were virtually absent in the platelets after circulation. The adhesion to collagen and platelet aggregation after circulation was significantly reduced.
Bartoli et al. [29]	N = 14 whole blood (human)	CPDA-1	TORVAD, HMII	6 h (0, 6 h)	- (-)	ELISA (plasma CD41/61)	The TORVAD did not activate platelets whereas the HMII caused significant platelet activation (P < 0.001)
Gandini et al. [30]	N = 10 whole blood (human)	Citrate (circuits coated with 400 IU of heparin or 20 μM IMB-F12)	Rotaflow centrifugal pump	6 h (every 2 h)	Streck cell preservative (FACS lysing solution)	Flow cytometry (CD62P, CD42b), Luminex multiplex immunoassays (PF4), Impedance aggregometry	CD62P and CD42b were stable, whereas PF4 decreased over time. Platelet aggregation was significantly decreased over time (P < 0.001).
Hioki et al. [31]	- whole blood (human)	-	Roller pump (unclear)	3 h (0, 5 min, 1 h, 2 h, 3 h, and 2 hours after cessation of circulation)	- (-)	Flow cytometry (CD42b), Platelet aggregation method (unclear)	Level of CD42b decreased on initiating circulation. The extent of shear stress induced platelet aggregation significantly decreased during circulation.
Horobin et al. [32]	N = 7 whole blood (human)	CPDA-1 (samples with EDTA or citrate)	HVAD	5 h (0, 15, 45, 90, 180, 300 min)	- (-)	Impedance aggregometry	ADP- and collagen-induced platelet aggregation significantly decreased after 5 h (P < 0.01; P < 0.05). Ristocetin-induced platelet aggregation significantly increased after 45 min (P < 0.05).
Stohlawetz et al. [33]	N = 12 whole blood (human)	CPDA-1 / heparin (samples with hirudin)	HVAD	2 h (0, 15, 30, 60, 120 min)	- (-)	Impedance aggregometry	Platelet function was impaired during circulation. Aggregation tests in response to four different agonists significantly and markedly decreased after the extracorporeal circulation (P < 0.005).
Kawahito et al. [34]	N = 5 whole blood (human)	Heparin	Capiox centrifugal pump	3 h (every 1 h)	- (-)	ELISA (β-TG)	β-TG levels increased over time.
Kawahito et al. [35]	- whole blood (human)	Heparin	Capiox centrifugal pump	3 h (every 1 h)	- (-)	ELISA (β-TG, PF4)	β-TG and PF4 increased over time. The rates of increase in β-TG and PF4 for the high-pressure head condition demonstrated significantly higher values than those in the low-pressure head condition.
King et al. [36]	N = 10 whole blood (human)	2.5 U/mL heparin	Medtronic centrifugal pump	72 h (0, 10, 30 min, 1, 2, 3, 4, 5, 6, 8, 12, 24, 48, 72 h)	- (-)	ELISA (PF4)	PF4 levels steadily increased to 6.12 times the baseline value over 72 hours of in-vitro circulation.
Linneweber et al. [37]	N = 5 whole blood (human)	1 U/mL heparin	Modified Baylor Gyro 710 VAD	2 h (0, 5, 15, 30, 60, 120 min)	- (-)	ELISA (CD42b), SEM	Under high-shear levels (>2500 [/s], impeller speed of 3500 rpm) or low-shear levels (>200 [/s], impeller speed of 1500 rpm), platelet adhesion reached above 95% on the impeller. Activated platelets changed extensively in morphology on all examined impellers under SEM.
Zayat et al. [38]	N = 8 whole blood (human)	3.75 U/mL heparin	HM3, EVAHEART	6 h (every 1 h)	BD Cell Fix (-)	Flow cytometry (CD62P, CD61)	With the optimal settings, no significant increase in CD62P expression was observed between baseline and 360 mins. In contrast, under equal settings, CD62P expression increased significantly between baseline and 360 mins (EVAHEART: P ¼ .006; HM3: P ¼ .012).
Musial et al. [39]	N = 6 whole blood (human)	5 U/mL heparin (samples with citrate, EDTA or ACD)	Roller pump (unclear)	2 h (5, 30,60, 120 min)	- (-)	Lumi-aggregometry, Radioimmunoassay (β-TG), Western blotting (CD61), SEM	β-TG antigen level in the plasma during simulated extracorporeal circulation increased overtime. In the absence of protective agents, a high concentration of CD61 was present. Control platelets showed a characteristic increase in light transmission.
Chan et al. [40]	N = 12 whole blood (bovine)	CPDA-1	HVAD	6 h (every 2 h)	Streck solution (-)	Flow cytometry (CD41/61)	The level of platelet activation detected by CD41/61 remained the same overtime for all conditions.
Chan et al. [41]	N = 7 whole blood (bovine)	CPDA-1	CentriMag	6 h (every 1 h)	BD stabilizing fixative (BD FACS lysing solution)	Flow cytometry (CD41/61, unknown antigen)	No significant change of platelet activation was observed in CentriMag after 6 hours. There was no significant difference between CentriMag and the static control for platelet activation (P ≥ 0.9).
Furugaki et al. [42]	N = 9 whole blood (human)	4 U/mL heparin (samples with citrate)	Stokert roller pump	30 min (0, 5, 10, 20, 30 min)	- (-)	Laser-light scattering assay, Flow cytometry (CD42b, CD62P, PS)	Platelet aggregation gradually decreased after 30 min of circulation (P < 0.001). The mean surface PS exposure percentage rose slightly higher during recirculation (P > 0.05). CD62P rose slightly higher compared to before circulation (P > 0.05).
Linneweber et al. [43]	N = 6 whole blood (human)	1 U/mL heparin	Sarns roller pump, Gyro centrifugal pump	2 h (0, 2 h)	1 % PFA (-)	Flow cytometry (CD42b)	Platelet aggregation increased after 2 hours in both pumps. Significantly more platelet aggregates and platelet consumption in the roller pump than in the centrifugal pump (P < 0.01).
Mellgren et al. [44]	N = 8 whole blood (human)	0.56 U/mL heparin (samples with EDTA, heparin, or citrate)	Polystan roller pump, Medtronic BP-50	24 h (0, 0.5, 1, 3, 12, 24 h)	1 % PFA (-)	Flow cytometry (CD42b, CD61), ELISA (β-TG, platelet serotonin content, plasma glycocalicin)	Platelet membrane CD42b expression displayed a progressive and pronounced decrease over the entire 24-hour perfusion experiment. A reduction in the platelet membrane CD61 expression was also observed, but to a considerably lesser extent compared with CD42b. A significant increase of plasma β-TG concentration and a decrease of intraplatelet serotonin was observed. The plasma concentration of glycocalicin increased in parallel with a reduced platelet surface expression of CD42b.
Meyer et al. [45]	N = 5 whole blood (porcine)	4 U/mL heparin	4 ECMO systems	6 h (every 2 h)	- (-)	Flow cytometry (CD61)	None of the ECMO systems had a significant impact on platelet aggregation above baseline.
Mohara et al. [46]	N = 5 whole blood (human)	Heparin	Medtronic BP-80, Capiox centrifugal pump	3 h (every 1 h)	- (-)	ELISA (β-TG, PF4)	The rates of increase in β-TG and PF4 levels rose in both pumps after 3 h but the increases observed in the Capiox were significantly lower than those in the BP-80 ( P < 0.05).
Rinder et al. [47]	N = 4 whole blood (human)	5 U/mL heparin	CI roller pump	90 min (0, 5, 15, 30, 45, 60, 75, 90 min)	1 % PFA (-)	Flow cytometry (CD41/61, CD62P)	The percentage of CD62P-positive platelets increased significantly during control simulated extracorporeal circulation after 90 minutes of recirculation (P = 0.01).
Sheriff et al. [48]	N = 15 GFPs (human)	Aspirin	DeBakey	30 min (0, 10, 20, 30 min)	- (-)	PAS assay	Normalized PAS and platelet activation rate increased in all circuits after 30mins. Platelets exposed to aspirin in vitro showed reduction in platelet activation rate, compared to untreated controls.

HMII, HeartMate II; GPVI, Glycoprotein VI; MFI, Mean fluorescence intensity; PFA, Paraformaldehyde; HM3, HeartMate 3; vWF, von Willebrand Factor; GFPs, Gel-filtered platelets; ACD-A, Anticoagulant citrate dextrose solution A; PS, Phosphatidylserine; PAS, Platelet activity state

CPDA-1, Citrate phosphate dextrose adenine-1; ELISA, Enzyme-linked immunosorbent assay; β-TG, β-Thromboglobulin; EDTA, Ethylenediaminetetraacetic acid; BD, a company name; FACS, Fluorescence-activated cell sorting

SEM, Scanning electron microscope; ECMO, Extracorporeal membrane oxygenation

SFP, Screen filtration pressure; PATI, Platelet aggregation threshold index; TEM, Transmission electron microscopy

IMB-F12, A kind of anticoagulant; PF4, Platelet factor 4; ADP, Adenosine diphosphate

## Blood Sources

Whole blood was the primary experimental material in 35 studies, with human samples most frequently used (*n* = 27), followed by porcine (*n* = 4) [15, 23, 25, 45] and bovine (*n* = 4) [16, 20, 40, 41]. In contrast, four studies employed gel-filtered platelets (GFPs), of which three used human GFPs [14, 26, 48] and one used bovine GFPs [27]. The number of donors varied substantially across studies, ranging from 1 to 17, with the majority falling between 5 and 10. Notably, two studies did not report their number of donors [31, 35].

## Experiment Duration and Sampling Strategy

Among the 39 studies, most experimental durations ranged between 3 and 6 hours (*n* = 26). Eleven studies reported shorter exposure times of 2 hours [27, 33, 37, 39, 43], 1.5 hours [47], 1 hour [14, 25], and 30 minutes [26, 42, 48], respectively, while two studies extended to 24 hours [44] and 72 hours [36], respectively. Sampling frequency varied across studies: thirteen collected samples every hour [10–13, 17, 18, 21, 22, 34, 35, 38, 41, 46], five every two hours [20, 30, 40, 43, 45], and one every three hours [23]. Roka-Moiia et al. conducted frequent sampling, collecting every 30 minutes [15]. In two studies, samples were collected every 10 minutes [26, 48]. In the 24-hour study, samples were collected at multiple time points (0, 0.5, 1, 3, 12, and 24 hours) [44]. The remaining studies followed uncommon sampling intervals.

## Anticoagulant

Citrate was the most commonly employed anticoagulant to prevent clot formation after whole blood collection, reported in 22 studies. The formulations included 3.8% sodium citrate (*n* = 4) [10, 12, 18, 21], 3.2% sodium citrate (*n* = 4) [11, 13, 22, 25], unspecified sodium citrate (*n* = 3) [17, 26, 30], acid citrate dextrose solution A (ACD-A, *n* = 1) [14], and citrate phosphate dextrose adenine−1 (CPDA-1, *n* = 10) [15, 16, 19, 20, 27, 29, 32, 33, 40, 41]. Some studies, however, deviated from this standard practice. For example, Oota-Ishigaki et al. anticoagulated blood with 3.2% sodium citrate but subsequently added 20 mL of 2% calcium chloride to reverse citrate’s effects prior to experimentation, while also supplementing the blood with 4 mL of heparin to further prevent clotting [25]. In another study, blood was anticoagulated with sodium citrate, and the experimental circuits were coated with either 400 IU of heparin or 20 μM IMB-F12 [30]. Roka-Moiia et al. reported pre-anticoagulation of porcine with systemic heparin (200 U/kg) prior to vessel catheterization, followed by blood collection into bags containing CPDA-1 [15]. Furthermore, in three studies, blood was anticoagulated with CPDA-1, however, samples designated for platelet function measurements were subsequently transferred into tubes containing ethylenediaminetetraacetic acid (EDTA), citrate, or hirudin [19, 32, 33].

Heparin was utilized as the anticoagulant in 15 studies. In 11 of these, the reported concentration ranged from 0.56 U/mL to 5 U/mL; notably, four studies also collected samples into tubes containing citrate, EDTA, ACD, or heparin [23, 39, 42, 44]. In four additional studies, the use of heparin was reported without specification of concentration [24, 34, 35, 46]. One of these studies further compared different anticoagulants, including tirofiban, apyrase, and EDTA, alongside heparin [24].

In addition to conventional anticoagulants, one study used aspirin to prevent clot formation [48], while another did not disclose the anticoagulant employed [31].

## MCS Devices and Mock Loops

Across 39 studies, blood or GFPs were exposed to MCS devices within mock-circulation loops. Among these, centrifugal pumps were the most commonly employed (*n* = 28), followed by axial-flow pumps (*n* = 9) and roller pumps (*n* = 10) as shown in Table 1. Additionally, one study utilized a toroidal-flow pump (TORVAD) [29], and another employed a volume-displacement pump (Berlin Heart Excor) [20]. The configuration of mock loops in all studies included in this review is summarized in Table 2. Loop volumes ranged widely from 60 mL [28] to 850 mL [20], reflecting differences in experimental objectives and device scales. Reservoir designs also showed considerable variability in shape and size, with three studies explicitly reporting the absence of a reservoir [37, 43, 48]. Similarly, tubing materials and dimensions differed across studies, with PVC and silicone tubing most commonly used, typically featuring internal diameters of approximately 3/8 inch, but occasionally ranging from 1/4 to 1/2 inch. In addition, seven studies reported the details of oxygenators used in their experimental setups [13, 27, 28, 39, 44, 45, 47], and two studies incorporated mesh filters to remove clots generated within the loop [15, 25].

**Table 2:** The configuration of mock loops across the included studies of this review

Reference	Loop volume ( mL)	Reservoir	Tube	Oxygenator	Clamp / Flow resistor	Heater	Additional
Zhang et al. [10]	450	a heat–sealed blood bag	–	no	a flat clamp	no	–
Wang et al. [11]	300	a blood bag	PVC tubing(3/8–inch ID)	no	a Hoffman clamp	no	–
Tarafder et al. [12]	300	a blood bag	PVC tubing(3/8–inch ID)	no	a Hoffman clamp	no	–
Sun et al. [13]	300	a custom–made soft–shell bag	(3/8–inch ID)	A pediatric oxygenator	yes	–	–
Roka–Moiia et al. [14]	–	–	non–thrombogenic Tygon tubing(1/2–inch ID)	no	–	–	–
Roka–Moiia et al. [15]	750	–	Tygon tubing(1/2–inch)	no	yes	water bath	a mesh filter
Radley et al. [16]	450	–	–	–	–	water bath	–
Chen et al. [17]	300	a soft–shell blood bag	(3/8–inch)	no	yes	no	–
Chen et al. [18]	> 450	a small–volume blood bag	(3/8–inch)	no	yes	no	–
Chan et al. [19]	185	reservoirs (R–38, Medtronic)	Medtronic tubing (∅ 9.5 mm)	no	a Hoffman clamp / a 3D–printed restrictor	water bath	–
Chan et al. [20]	850	–	–	–	a custom–made clamp	no	–
Berk et al. [21]	300	a sealed blood bag	Tygon tubing (3/8–inch)	no	a flat clamp	no	–
Tanaka et al. [22]	90	–	PVC tubing(3/8–inch)	no	a resistance tubing(1/4–inch ID)	water bath	–
Roberts et al.[23]	–	yes	PVC tubing(3/8–inch ID, 1 m length)	no	–	a Hemotherm® heater	–
Rauch et al. [24]	–	–	–	yes	–	–	–
OotaIshigaki et al. [25]	400	a triangular soft reservoir	–	no	–	water bath	a 150–μm micromesh filter
Girdhar et al. [26]	120	–	Tygon R3603 tubing(1/2–inch ID)	no	Tygon R3603 flow resistor tubing(1/4–inch ID)	–	–
Consoloet al. [27]	95	a 50–mL Falcon tube	–	a polypropylene hollow fiber oxygenator ( 0.22m^2^)	–	water–bath	–
Arao et al. [28]	60	–	VC tubing (6 mm ID, 60 cm length )	an Oxia IC–N membrane oxygenator	–	–	–
Bartoli et al. [29]	500	a reservoir(1L)	Tygon tubing(3/8–inch)	no	a c–clamp resistor	water basin	–
Gandini et al. [30]	–	a blood bag	–	yes	a Hoffman clamp	yes	–
Hioki et al. [31]	–	yes	–	no	no	no	–
Horobin et al. [32]	225	a reservoir (R–38, Medtronic)	tubing (9.5 mm ID)	no	a vice–grip clamp	yes	–
Stohlawetz et al. [33]	90	a thin–walled silicone tubing (3/8–inch, 18 cm length, 0.6 mm thickness)	silicone tubings / PVC tubing (Tygon® S50 HL)	no	a resistance clamp	incubator	–
Kawahito et al. [34]	95	yes	PVC tubing(3/8–inch)	no	resistance tube (1/4–inch ID, 27 cm length)	water bath	–
Kawahito et al. [35]	92 /125	yes	PVC tubing(3/8–inch)	no	resistance tube (1/4–inch ID, 27 cm length )	water bath	–
King et al.[36]	450	a PVC venous reservoir bag	PVC tubing	no	no	water bath	–
Linneweber et al. [37]	300	no	PVC tubing (3/8–inch)	no	–	–	–
Zayat et al. [38]	82 / 100	yes	PVC tubing (3/8–inch, 1/4–inch, 1/16–inch)	no	a hose clip	heating hood	–
Musial et al. [39]	300	a PVC reservoir bag	silicone rubber tubing (3/8–inch ID)	a spiral coilmembrane oxygenator (model 0400–2A, 0.4m^2^)	–	water bath	–
Chan et al. [40]	450	a custom–made blood reservoir	E–3603 Tygon tubing (Ø 9.5 mm / Ø 12.7 mm)	no	a resistance clamp	water bath	–
Chan et al.[41]	450	yes	–	–	a custom–made clamp	water bath	–
Furugaki et al. [42]	100	a soft bag reservoir (50 mL)	VC tubing (6 mm ID, 60 cm length)	–	–	constant 25 °C	–
Linneweber et al. [43]	150	no	PVC tubing (3/8–inch)	–	an adjustable throttle	–	–
Mellgren et al. [44]	–	a bladder box	–	a membrane oxygenator	–	yes	–
Meyer et al. [45]	–	a Gish reservoir bag	1/4–inch heparin– coated tubing	a hollow–fiber oxygenator / silicone membrane oxygenator	an arterial catheter	yes	–
Mohara et al.[46]	95 / 130	yes	PVC tubing(3/8–inch)	no	resistance tubing (1/4–inch ID, 27 cm length)	water bath	–
Rinder et al.[47]	700	–	–	a pediatric membrane oxygenator	–	yes	–
Sheriff et al. [48]	–	no	Tygon R3603 loop tubing (1/4–inch ID)	no	Tygon R3603 flow resistor tubing (47–inch length)	yes	–

PVC, polyvinyl chloride; ID, inner diameter; VC, vinyl chloride. – means not mentioned in the original study

## Platelet Function Evaluation Method

All methods used to evaluate platelet function are listed in Table 3. Detailed results of most widely used methods of flow cytometry and Enzyme-linked immunosorbent assay (ELISA) are followed below.

**Table 3:** Summarization of platelet function evaluation methods in this review

Platelet function	Methods (Number of studies)
Platelet activation	Flow cytometry (n = 25) ELISA (n = 9) Luminex multiplex immunoassays (n = 1) Radioimmunoassay (n = 1) Western blotting (n = 1)
Platelet aggregation	Laser-light scattering assay (n = 2) Optical density method / Light transmission assay (n = 2) Impedance aggregometry (n = 4) Lumi-aggreometry (n = 1) Screen filtration pressure aggreometry (n = 1) Unclear (n = 1)
Platelet adhesion	Fluorescent microscope (n = 2) Photographed image (n = 1)
Platelet clot	Transmission electron microscopy (n = 1) Scanning electron microscope (n = 4)
Platelet activity	PAS (n = 4)

ELISA, enzyme-linked immunosorbent assay; PAS, platelet activity state assay

## Flow Cytometry

### Sample Fixation

A total of eighteen studies reported the use of chemical reagents for platelet fixation during sample processing. The most commonly used fixative was paraformaldehyde (PFA, *n* = 11), followed by Streck Cell Preservative solution (*n* = 3) [16, 30, 40], BD Stabilizing Fixative solution (*n* = 3) [19, 20, 41], and BD Cell Fix (*n* = 1) [38]. Among the PFA-based protocols, five studies employed 1% PFA for 30 minutes at 4 °C in the dark [11, 13, 17, 18, 21], two used 1% PFA for 1 hour at room temperature [15, 47], and one utilized 3.5% PFA for 20 minutes [14]. Three studies mentioned PFA without more detailed information [28, 43, 44]. For Streck Cell Preservative solution, one study fixed platelets by gently inverting samples ten times after adding 100 µL of the solution in 100 µL blood, followed by overnight storage at 4 °C [16], while two studies used a 1:1 volume ratio of fixative and sample, with measurements conducted immediately thereafter [30, 40]. Regarding BD Stabilizing Fixative solution, two studies employed 200 µL in total without specifying further details on processing conditions [19, 20], and one stored fixed samples at 4 °C overnight before measurement [41]. BD Cell Fix was applied in one study prior to fluorescence-activated cell sorting (FACS) analysis [38].

## Lysis Buffer

The use of lysis buffer during sample preparation was reported only in 5 studies. Four studies used 2 mL of BD FACS Lysing Solution to remove red blood cells [19, 20, 30, 41], one study used BD lyse wash assistant instrument to lyse red blood cells and remove unbound antibodies [23].

## Sample Processing Strategy

Sample processing procedures varied considerably across the studies as shown in Table 4.

**Table 4:** Summarization of sample processing procedures for flow cytometry in the review

Sampling-Staining-Fixation-Analysis	[11, 13, 17, 18, 21, 38, 40, 43]
Sampling-Staining-Fixation-Washing-Analysis	[14]
Sampling-Staining-Lysis-Washing-Analysis	[23]
Sampling-Staining-Lysis-Washing-Fixation-Overnight Storage-Analysis	[19, 20]
Sampling-Staining-Washing-Analysis	[45]
Sampling-Staining-Washing-Lysis-Washing-Fixation-Overnight Storage-Analysis	[41]
Sampling-Fixation-Staining-Overnight Storage-Analysis	[16]
Sampling-Fixation-Staining-Lysis-Washing-Analysis	[30]
Sampling-Fixation-Staining-PRP Obtaining-Analysis	[42]
Sampling-Fixation-Washing-Staining-Washing-Analysis	[47]
Sampling-PRP Obtaining-Staining-Analysis	[15, 24, 28]
Sampling-PRP Obtaining-Staining-Fixation-Analysis	[44]
Sufficient procedural details were not provided	[10, 12, 31]

## Biomarkers

**Fig. 2:**
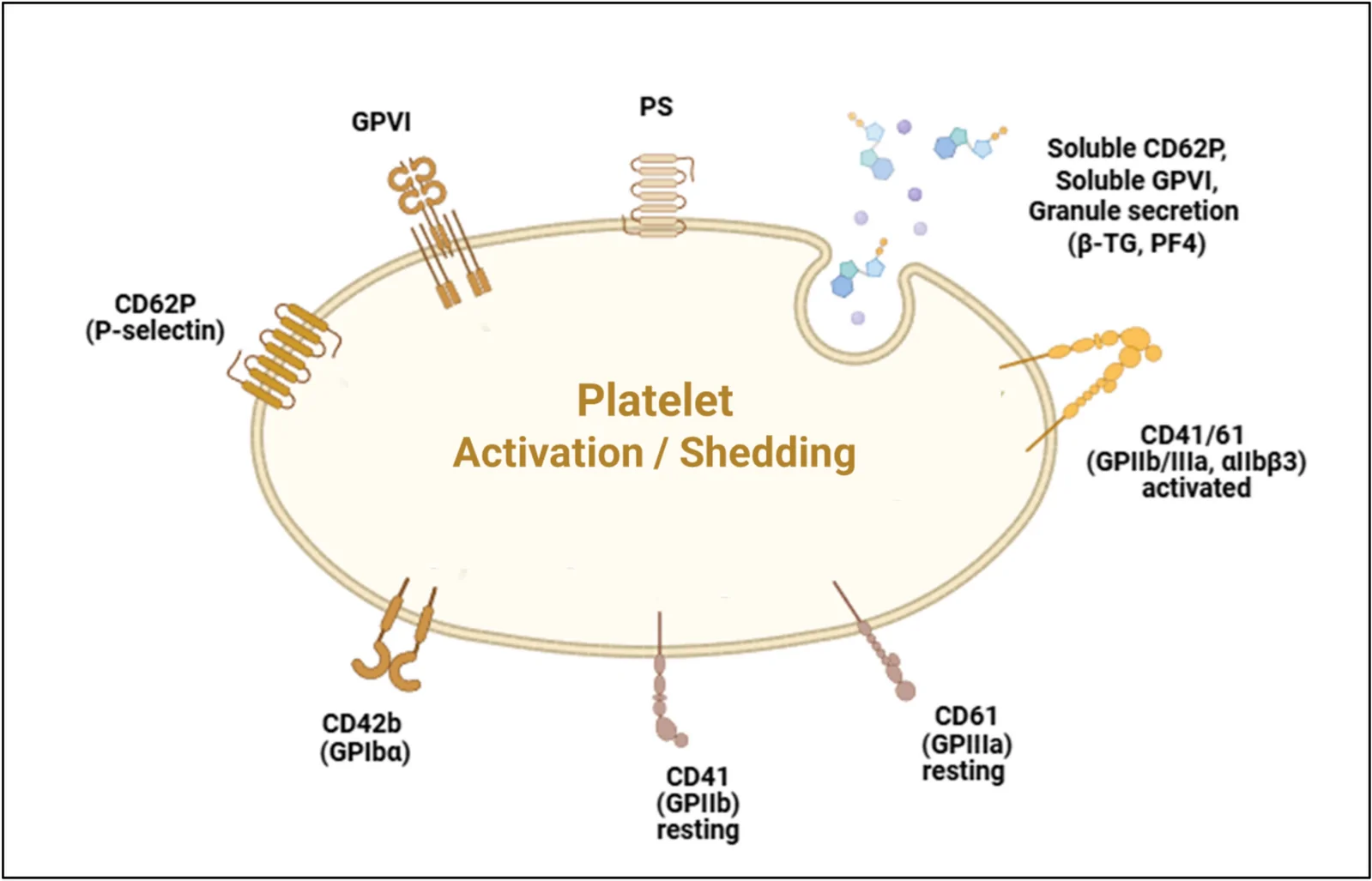
Summarization of the platelet markers assessed across the included studies using flow cytometry and ELISA test in this review. PS: Phosphatidylserine, β-TG: β-Thromboglobulin, PF4: Platelet factor 4

The biomarkers detected under flow cytometry across the included studies of this review are presented in Fig. 2 and the effect of MCS devices on these biomarkers are listed in Table 5 below.

**Table 5:** Summarization of biomarkers detected via flow cytometry in the review

Biomarkers	Species	Expression of biomarkers after circulation
CD41/61 (GPIIb/IIIa, αIIbβ3) (n = 11)	Human (n = 7)	Percentage of positive platelets increased [17, 18, 21] Percentage of positive platelets decreased [11] Fluorescence intensity of positive platelets increased [10] Fluorescence intensity of positive platelets decreased [13] Percentage and fluorescence intensity of positive platelets were stable [14]
	Bovine (n = 4)	Percentage of positive platelets increased [16, 20] Percentage of positive platelets were stable [40, 41]
CD62P (P-selectin) (n = 10)	Human (n = 8)	Percentage of positive platelets increased [19, 28, 38, 47] Percentage and fluorescence intensity of positive platelets mildly increased [14] Fluorescence intensity of positive platelets was stable [30] Percentage of positive platelets was stable [42] Level of positive platelets increased [12]
	Porcine (n = 2)	Fluorescence intensity of positive platelets decreased [15] Percentage of positive platelets increased [23]
CD42b (GPIbα) (n = 13)	Human (n = 13)	Percentage of positive platelets increased [19, 43] Fluorescence intensity of positive platelets decreased [10, 13, 17, 18, 24, 28, 31, 44] Fluorescence intensity of positive platelets was stable [30] Level of positive platelets decreased [12] Used to identify platelet population [42]
CD41(GPIIb) (n = 9)	Human (n = 9)	Used to identify platelet population [11, 13, 14, 17, 18, 21, 30, 47] Fluorescence intensity of positive platelets decreased [28]
CD61 (GPIIIa) (n = 4)	Human (n = 2)	Used to identify platelet population [38] Fluorescence intensity of positive platelets decreased [44]
	Porcine (n = 2)	Used to identify platelet population [23] Percentage of positive platelets was stable [45]
GPVI (n = 6)	Human (n = 6)	Fluorescence intensity of positive platelets decreased [10, 13, 17, 18, 24] Level of positive platelets decreased [12]
PS (n = 5)	Human (n = 3)	Percentage and fluorescence intensity of positive platelets increased [14] Percentage of positive platelets increased [28] Used to identify platelet population [42]
	Porcine (n = 2)	Percentage of positive platelets increased [23] Used to detect membrane depolarization and the percentage of positive microparticles increased [15]

GPVI, glycoprotein VI; PS, phosphatidylserine

### ELISA

Among the nine studies utilizing ELISA, two measured soluble CD62P (P-selectin). Roka-Moiia et al. found undetectable levels in porcine plasma after four hours of circulation [15], whereas human plasma showed significant increases after two hours [18]. Five studies assessed β-Thromboglobulin (β-TG), of which four reported time-dependent increases [34, 35, 44, 46], while one found no change in porcine samples after circulation [15]. Three studies observed elevated platelet factor 4 (PF4) levels during circulation [35, 36, 46]. Additional findings included decreased intracellular serotonin and increased plasma glycocalicin (the glycosylation of CD42b) with reduced CD42b expression [44]. Bartoli et al. reported a significant increase of plasma CD41/61 in HM2 but no change in TORVAD [29] and Linneweber et al. noted platelet adhesion (activation) reaching above 95% in mock loops after circulation [37].

## Discussion

This integrated review provides a comprehensive summary of in vitro studies investigating platelet function in blood-contacting MCS devices. Across the 39 studies we included, substantial heterogeneity was observed in experimental materials, anticoagulation strategies, device types, and platelet function evaluation methods. While such diversity reflects the broad methodological nature of this field, it simultaneously underscores the lack of standardized testing protocols, making cross-study comparison challenging and limiting the interpretability of platelet responses to mechanical shear force.

## Variability in Blood Sources

Human whole blood was the predominant sample type, reflecting its physiological relevance and translational value. Nevertheless, animal blood, particularly porcine and bovine, was also frequently used due to its accessibility and ethical considerations. Although these models enable large blood volume collection and controlled experimentation, species-specific differences in platelet size, reactivity, and coagulation properties may influence shear-induced responses and partly explain inconsistencies in activation markers across studies. The comparative work by Chan et al. [49], exemplifies the value of multispecies analyses, however, such systematic evaluations remain the exception. In addition, several studies employed GFPs, which provide valuable mechanistic insights on platelet behavior but limited physiological comparability due to reduced viscosity and absence of other blood components [50–53]. Together, these findings highlight the need to balance experimental feasibility with physiological relevance when choosing blood sources and sample preparation.

## Anticoagulation Strategies as a Major Preclinical Variable

Citrate-based anticoagulants (sodium citrate, ACD-A, CPDA-1) were most commonly used across studies. While citrate effectively prevents clotting by chelating extracellular calcium, differences in citrate formulations, including calcium-binding capacity, buffering properties, and glucose content, may alter platelet activation thresholds [54, 55]. Importantly, calcium chelation can blunt shear-induced activation, leading to possible underestimation of platelet responsiveness. Efforts to counteract this effect through recalcification or supplemental heparin introduce further variables that complicate interpretation [14, 25–27, 48]. Heparin was also used at different concentrations ranging from 0.56 to 5 U/mL. Despite its clinical familiarity, the in vitro effects of heparin on platelet function remain poorly characterized. As reported, heparin can interfere with platelet function assays [56] and, in some cases, enhance expression of platelet activation markers [57]. However, the extent to which the choice of anticoagulant influences biomarker expression remains unclear, thereby limiting the interpretability of comparative analyses. Overall, anticoagulant choice represents a critical yet often underappreciated pre-analytical variable in vitro MCS research.

## Platelet Function Assessment Methods

A wide range of analytical techniques has been employed to investigate and evaluate platelet function in in vitro studies involving MCS devices, however, weak concordance between methods underscores the complexity of capturing shear-dependent platelet responses.

Flow cytometry was the most frequently used method, due to its sensitivity and ability to distinguish specific activation pathways with low platelet counts [58, 59]. Recent evidence suggests that supraphysiological shear stress paradoxically reduces CD41/61, CD62P and GPIb expression [60], likely reflecting activation followed with subsequent receptor shedding or receptor shedding alone. This aligns with a few studies reporting decreased fluorescence intensity for CD42b and GPVI in sheared samples [10, 13, 17, 18, 24]. In contrast, findings for CD41/61 were inconsistent, stemming from differences in marker selection, fixation procedures, and sampling protocols [61–63]. Notably, Roka-Moiia et al. reported that continuous shear stress did not induce CD41/61 activation or CD62P exposure, whereas biochemical agonists readily triggered both responses [14]. These findings indicate that supraphysiological shear stress induces a distinct platelet activation pattern compared with biochemical stimulation, underscoring the need to further investigate the mechanistic differences between these pathways.

A recent review indicates that shear-induced platelet activation in microenvironments is primarily mediated through the PI3K/Akt, downstream of mechanosensitive signaling pathway, such as vWF-GPIb-IX-V receptor complex or Piezo1, with involvement of receptors such as CD62P, GPIb–IX, GPIIb/IIIa, GPVI, and αIIβ1 integrin [64]. However, the shear conditions encountered by platelets in microenvironments may not reflect those in mock-loop studies. Therefore, shear-induced platelet responses observed in microenvironmental warrant validation under controlled mock-loop conditions in future studies. These observations also raise a further important question: to what extent does shear exposure alter platelet’s subsequent responsiveness to biochemical agonists? Clarifying this will be essential for advancing our understanding of shear-mediated platelet dysfunction in MCS environments.

Additionally, a precise and standardized characterization of platelet biomarkers in flow cytometry analyses is essential to ensure comparability across studies. In this context, the most commonly reported parameters were mean fluorescence intensity (MFI) and the percentage of positive platelets. MFI reflects relative receptor density and may indicate receptor shedding under high shear conditions, whereas the percentage of positive platelets represents the proportion of platelets expressing a given marker. However, reliance solely on the percentage of positive platelets is problematic for constitutively expressed receptors, such as GPVI or GPIb, where baseline expression is present on nearly all platelets; therefore, concurrent reporting of both MFI and percentage positivity is recommended to provide a more comprehensive and accurate assessment of platelet activation and phenotype.

Ten studies examined platelet aggregation using a range of techniques, including laser-light scattering assays [22, 42], impedance aggregometry [23, 30, 32, 33], light transmission aggregometry [22, 28] among others. However, substantial methodological variation, particularly in protocols and agonist selection limits comparability across studies. Moreover, aggregometry provides information only on a platelet’s capacity to respond to exogenous agonists and does not directly reflect the underlying activation state induced by shear exposure.

Platelet adhesion was quantified in three studies using diverse approaches, including fluorescent microscopy [13, 28] and subsequent image analysis software (ImageJ) [25]. Although these methods enable visualization and quantification of platelet-surface interactions, the lack of standardized substrates and flow conditions limits direct comparability across investigations.

Electron microscopy techniques were used in a subset of studies to assess platelet morphology and thrombus formation under shear conditions. Scanning electron microscopy (*n* = 4) [23, 25, 37, 39] and transmission electron microscopy (*n* = 1) [28] provided detailed visualization of platelet adhesion, aggregation, and ultrastructural deformation. While these techniques offer qualitative insights into platelet-surface interactions and clot architecture, they are inherently limited by their low throughput and potential sample preparation artifacts.

Enzyme-linked immunosorbent assays (ELISAs), used in nine studies, were primarily employed to quantify soluble platelet activation markers such as β-TG, PF4, and soluble CD62P. While several studies reported time-dependent increases in β-TG and PF4, others observed minimal or no changes, particularly in non-human samples [15]. These discrepancies may arise from interspecies differences, variation in shear duration, or the influence of anticoagulants [49]. Additional soluble biomarkers, including serotonin, glycocalicin, and released CD41/61, provided complementary insights on platelet degranulation and secretion, but lack the cellular specificity of flow cytometry-based assays. Additionally, ELISA results are highly sensitive to plasma preparation methods, processing delays and storage conditions, all of which can introduce variability across studies. One study employing Luminex multiplex immunoassays demonstrated the potential for simultaneous quantification of multiple soluble activation markers, offering a higher-throughput and more integrative assessment of platelet activation pathways [30]. Similarly, one study utilized Western blotting to analyze platelet-derived proteins in plasma, providing insight into molecular alterations secondary to mechanical stress [39].

Four studies determined the procoagulant activity of platelets via the platelet activity state (PAS) assay [14, 26, 27, 48], which quantifies thrombin generation by GFPs and acetylated prothrombin (Ac-FIIa), a thrombin substrate in capable of inducing clot formation or secondary platelet activation. By providing near real-time measurement of thrombin production, the PAS assay effectively links mechanical shear exposure to procoagulant properties. However, the use of GFPs within a mock-loop limits its physiological relevance and comparability to in-vivo conditions.

## Device Diversity and Mock-Loop Configuration

Most studies focused on centrifugal pumps, followed by axial and roller pumps, with several investigating unconventional designs, such as toroidal-flow or volume-displacement pumps. Device geometry, flow characteristics, and operational settings directly affect shear exposure and even small divergences can produce divergent platelet responses. Notably, the currently available in vitro data on platelet responses are almost exclusively derived from continuous-flow LVAD platforms.

Emerging devices pose additional challenges for standardization. For example, total artificial hearts such as the BiVACOR, which incorporate two interconnected pumps [65], will require dedicated experimental setups and testing strategies, potentially limiting comparability across studies. Additionally, novel systems like the BiVACOR [65] and the CorWave LVAD [66] - featuring a biomimetic undulating polymer membrane - operate under pulsatile conditions in clinical use. The impact of such pulsatile flow on hemocompatibility remains an active area of research [67, 68] and is not adequately captured by the static mock circuits currently proposed in ASTM standards [69]. Moreover, variability in mock-loop configurations, including blood volume, tubing or device materials and circuit design, adds another layer of complexity. It has been reported that circulatory loop design and its components can introduce artifacts that influence the in vitro assessment of VAD thrombogenicity [70].

Accordingly, future preclinical assessments of hemocompatibility, particularly platelet function, should incorporate dynamic testing conditions that better replicate physiological hemodynamics. Despite the inherent heterogeneity of devices and mock-loop configurations, standardized reporting and harmonized experimental approaches will be essential to enable cross-study comparisons and to bridge the gap between preclinical findings and clinical outcomes.

## Clinical Relevance

To improve the clinical relevance of preclinical in vitro studies, we propose that future investigations adopt comparative study designs in which two or more devices are evaluated under identical experimental conditions and their relative effects are related to clinical outcomes. Direct comparison of absolute in vitro measurements with in vivo data are currently not feasible due to the limited availability of standardized datasets and the absence of validated thresholds linking biomarker levels to clinical risk.

In this review, only one in vitro study [12] directly compared platelet biomarkers between the HeartMate II (HMII) and HeartMate 3 (HM3), reporting higher CD62P expression in HMII alongside an approximately twofold reduction in CD42b and GPVI expression. These relative differences may align with clinical findings from the MOMENTUM 3 trial, where HMII was associated with higher rates of bleeding, stroke, and thrombotic events. Two additional in vitro studies [10, 21] reported comparable CD41/61 expression between HMII and HVAD pumps, which may reflect the broadly similar incidence of bleeding and thrombotic events observed in the ENDURANCE trial. However, the significantly higher stroke rate in the HVAD group (29.7% vs. 12.1%, P < 0.001) underscores that current biomarkers may not fully capture clinically relevant differences in device-related risk.

Further complexity is highlighted by clinical observations. For example, Smadja et al. [71] reported no significant changes in soluble CD40L, P-selectin, PF4, or NAP2 following pump implantation, with no differences between aspirin and heparin treatment groups. These findings suggest that commonly used platelet activation markers may not adequately reflect shear-induced platelet activation in vivo, potentially due to rapid clearance mechanisms or systemic regulatory processes. Alternatively, current mock-loop assays may overestimate platelet activation in devices with optimized shear profiles.

Overall, establishing meaningful links between in vitro findings and clinical outcomes requires a shift toward standardized, comparative preclinical study designs, combined with harmonized biomarker definitions and reporting. Such approaches may enable more robust cross-device comparisons and provide a more reliable bridge between preclinical testing and clinical performance. Nevertheless, in vitro models will likely remain inherently limited in their ability to predict clinical outcomes, as they cannot fully replicate complex physiological processes such as clearance mechanisms and systemic biological responses.

## Conclusion and Future Directions

Across the 39 studies reviewed, flow cytometry is the most utilized and promising method to evaluate the in vitro platelet function. Methodological heterogeneity emerged as the principal barrier to interpreting in vitro platelet testing in MCS research. Variations in sample type, anticoagulant use, fixation methods, sampling strategies, mock-loop configurations, and marker selection make cross-study comparison difficult and hinder the development of a coherent model of shear-mediated platelet activation. This variability also highlights a deeper gap: the mechanisms by which supraphysiologic shear alters platelet phenotype remain poorly defined. Although many observed responses, ranging from receptor shedding, phosphatidylserine exposure, impaired aggregation, and altered granule release, are well characterized in basic platelet biology, they are rarely thoroughly analyzed in MCS research in a standardized fashion.

To advance beyond descriptive findings, closer collaboration between engineering and platelet biology are required to determine whether supra-physiological shear induces true activation leading to aggregation or adhesion, apoptosis-like changes, functional exhaustion, or distinct mechano-stress-specific responses. A critical next step is establishing rigorous links between in vitro biomarkers and clinical events such as bleeding, thrombosis, acquired von Willebrand syndrome, or pump thrombosis. Pairing standardized in vitro tests with clinical datasets across specific devices and operating conditions will be key for translating bench findings into clinically meaningful predictors of hemocompatibility.

To advance the field, consensus guidelines such as ASTM should establish standardized mock-loop geometries, harmonized anticoagulation strategies, defined marker panels, validated sample processing protocols, consistent sampling time points, and comparative study designs. Such standardization would improve reproducibility, enable comparisons across studies and support regulatory acceptance of platelet-based assessments alongside conventional hemolysis testing.

## Data Availability

The authors confirm that all data generated or analyzed during this review are extracted form all included published articles.
